# Standardized exposure of the lateral and posterior wall in off-pump minimally invasive cardiac surgical coronary artery bypass grafting

**DOI:** 10.1016/j.xjtc.2024.06.002

**Published:** 2024-06-11

**Authors:** Alexander Albert, Georgi Petrov, Konstantinos Smiris, Philipp Angleitner

**Affiliations:** a Department of Cardiac Surgery, Klinikum Dortmund gGmbH, Dortmund, Germany; b Witten/Herdecke University, Witten, Germany; c Department of Cardiac Surgery, Medical University of Vienna, Vienna, Austria

**Figure:**
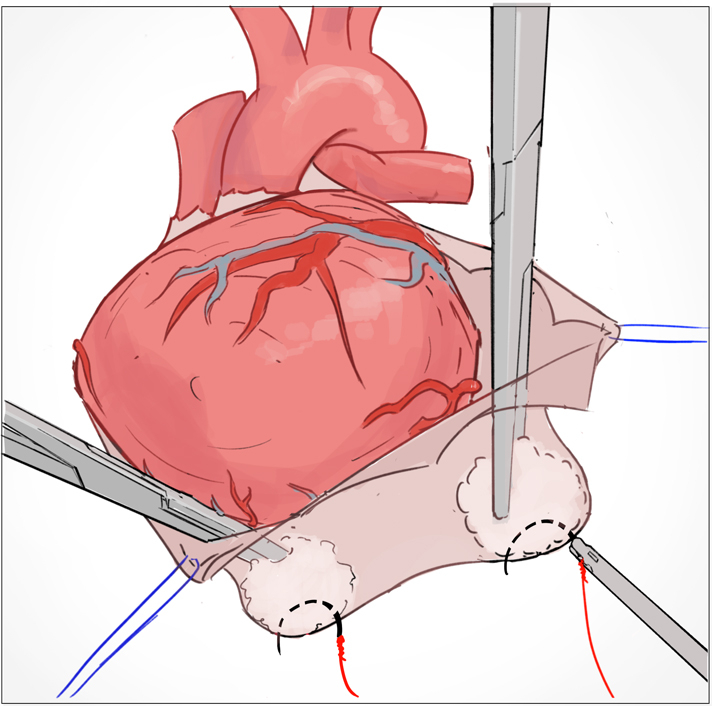
Standardized exposure of the lateral and posterior wall in off-pump MICS-CABG.

**Central Message**

We present a technique that overcomes the major obstacle for standardization and reproducibility of off-pump MICS-CABG: Exposure of coronary targets on the lateral and posterior wall.

Off-pump minimally invasive cardiac surgical coronary artery bypass grafting (MICS-CABG) involving multiple coronary targets is performed in limited numbers of centers, although long-term outcomes are excellent.^1^^,^^2^ Among the major challenges of off-pump MICS-CABG is adequate access to coronary targets on the lateral and posterior wall. A paucity of information has been published regarding specific techniques to accomplish this task in a standardized fashion. To attain visualization of the lateral and posterior wall, we developed the outside-inside technique based on the principle of bottom support as implemented in conventional off-pump coronary artery bypass grafting (OPCAB).^3^

According to our experience as an international training center for coronary revascularization, successful implementation and widespread adoption of surgical techniques requires deconstruction of procedures into individually teachable components.^4^ Our aim for this article was to specifically focus on the exposure and stabilization of the lateral wall in MICS-CABG. Institutional review board approval was received April 30, 2024 (vote # S-104/2024). Patients provided written informed consent.

## Surgical Technique

**Figure 1:**
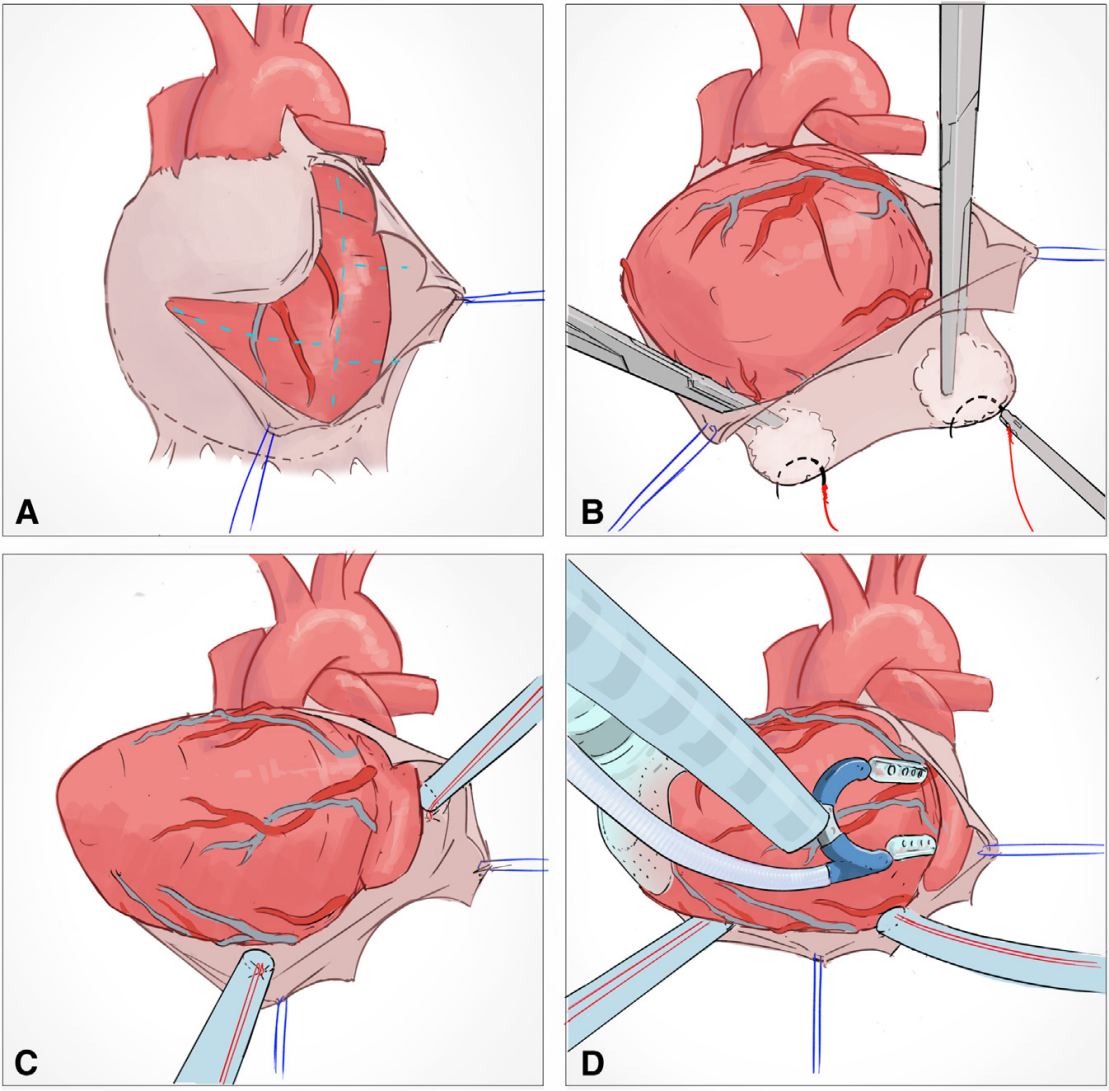
The outside-inside technique offers standardized exposure of lateral and posterior wall targets in off-pump minimally-invasive cardiac surgical coronary artery bypass grafting. Illustration: Karl Sokol.
Pericardial stitches: (Figure 1, *B*):
  • Diaphragmatic (to elevate the apex of the heart).
  • Through the left-lateral flap (to pull laterally and enable direct visualization of the lateral wall).
  • Along the total length of the lateral pericardium (to create a fan, allowing full-lung ventilation if necessary).^5^
Placement of 2 intrapericardial tourniquets (ie, the outside-inside technique) (Figure 1, *B*):
  • First: At the origin of the left lower pulmonary vein.
  • Second: Deeply posterior on the diaphragmatic aspect of the pericardium, close to the inferior caval vein.
Installation of apical suction device, if necessary (eg, Starfish; Medtronic) (Figure 1, *D*), followed by installation of a coronary stabilizer (eg, Octopus/Octopus Nuvo; Medtronic) (Figure 1, *D*).

Pericardial incisions (Figure 1, *A*):
  • Horizontally to the right (to enable enucleation of the heart).
  • Cephalad toward the pulmonary artery (to allow optimal course of the left internal thoracic artery).
  • Posteriorly to create a left-lateral flap (to allow a view on the lateral wall).

## Comment

Creation of bottom support for enucleation of the heart is a key element of conventional OPCAB. De Raet and colleagues^3^ have shown the importance of placing a pericardial sling deeply in the oblique sinus to concurrently elevate the left atrium and the left ventricle. The deeper the sling is anchored in the oblique sinus, the easier and safer the heart can be enucleated to visualize the lateral and posterior wall (according to the law of the lever). The technique by Yanagawa and Puskas^6^ proposes placement of tourniquets more inferiorly on the axis between the left lower pulmonary vein and the inferior caval vein, thereby creating a fold to elevate the heart. These methods have been demonstrated to be reproducible and teachable.^7^

However, these concepts are not directly translatable to MICS-CABG due to inaccessibility of the oblique sinus via minithoracotomy. For this purpose, we developed the outside-inside technique: The heart is elevated by 2 tourniquets and a pericardial fold between them (comparable to Puskas’ technique). One tourniquet is placed close to the left lower pulmonary vein, and a second tourniquet is placed as posteriorly and rightward as possible (below the phreno-pericardial ligament). In our experience, surgical complications caused by tourniquet placement have been rare. If sufficient exposure is not readily achieved by the outside-inside technique, we recommend leftward extension of the minithoracotomy as well as extension of the left lateral flap.

Generally, we believe that 2 aspects require specific attention to successfully implement off-pump MICS-CABG. First, we consider substantial experience in conventional OPCAB techniques and arterial revascularization prerequisites to off-pump MICS-CABG. With experience and the use of adequate surgical technique, conversion rates remain below 0.3%.^8^ Notably, MICS-CABG does not require complete enucleation of the heart. Frequently, exposure of the lateral and posterior wall is excellent even without the use of an apical stabilizer (Video 1). Our notion is that patients undergoing MICS-CABG remain more hemodynamically stable than patients undergoing conventional OPCAB. This aspect requires evaluation in a clinical trial.

Second, patient selection is essential, especially during the early learning curve. Four criteria have been considered contraindications against MICS-CABG: small coronary targets (eg, distal obtuse-marginal branches and distal posterior descending artery), left-ventricular ejection fraction <35%, cardiothoracic ratio > 0.5, and body mass index >30 (Video 1). In our experience, the outside-inside technique offers excellent exposure in most patients, thereby overcoming the difficulties related to these 4 conditions. In our practice, more patients have become suitable MICS-CABG candidates since introduction of the technique in May 2023.

## Conclusions

The outside-inside technique constitutes a successful modification of conventional OPCAB techniques to achieve similar grades of access to the lateral and posterior wall in MICS-CABG. We believe that this technique is an important contribution toward standardization, reproducibility, and wider adoption of MICS-CABG.

## Conflict of Interest Statement

Dr Albert is proctor for Medtronic and head of the Medtronic International Training Center for An-aortic OPCAB and MICS-CABG. All other authors have no conflicts of interest.

The *Journal* policy requires editors and reviewers to disclose conflicts of interest and to decline handling or reviewing manuscripts for which they may have a conflict of interest. The editors and reviewers of this article have no conflicts of interest.

## Supplementary Data

Video 1 The outside-inside technique in 2 patients undergoing off-pump MICS-CABG. Video available at: https://www.jtcvs.org/article/S2666-2507(24)00247-5/fulltext.
