# Supplementary figures and images for: Standardized exposure of the lateral and posterior wall in off-pump minimally invasive cardiac surgical coronary artery bypass grafting
Source: JTCVS Tech. 2024 Jun 11;26:61–3. doi: 10.1016/j.xjtc.2024.06.002 (PMC11329211; doi:10.1016/j.xjtc.2024.06.002)

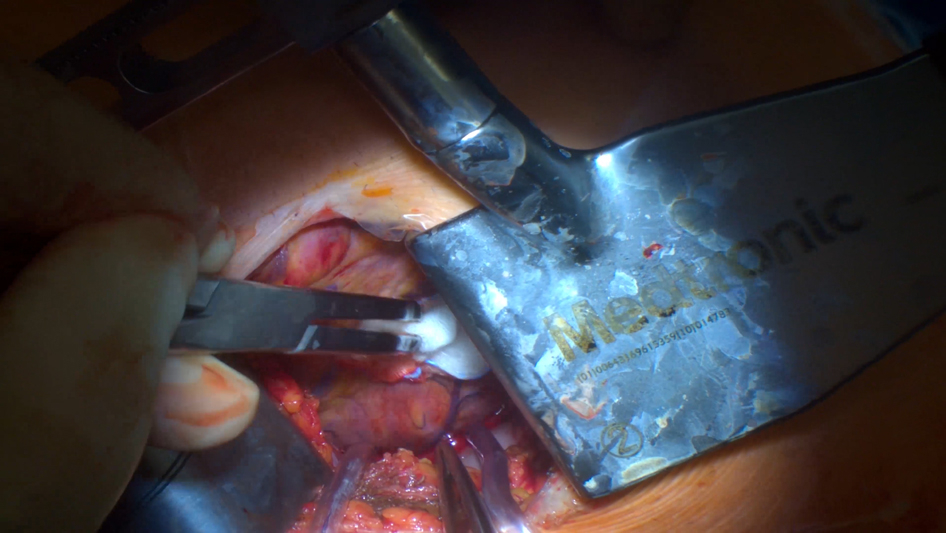

Supplement: Video 1 — The outside-inside technique in 2 patients undergoing off-pump MICS-CABG. Video available at: https://www.jtcvs.org/article/S2666-2507(24)00247-5/fulltext. [file fx2.jpg]
